# Whole genome mapping and identification of single nucleotide polymorphisms of four Bangladeshi individuals and their functional significance

**DOI:** 10.1186/s13104-021-05514-x

**Published:** 2021-03-20

**Authors:** Salim Khan, Shahina Akter, Barna Goswami, Ahashan Habib, Tanjina Akhtar Banu, Carl Barton, Eshrar Osman, Samiruzzaman Samir, Farida Arjuman, Saam Hasan, Maqsud Hossain

**Affiliations:** 1 Bangladesh Council of Scientific and Industrial Research, Dr. Kudrat-I-Khuda Road, Dhaka, 1205 Bangladesh; 2 Academica Solutions, London, WA1 1RG UK; 3 SciTech Consulting and Solutions, Dhaka, 1212 Bangladesh; 4 National Institute of Cancer Research Hospital, Dhaka, 1212 Bangladesh; 5 Department of Biochemistry and Microbiology, North South University, Dhaka, 1229 Bangladesh; 6 NSU Genome Research Institute (NGRI), North South University, Dhaka, 1229 Bangladesh

**Keywords:** Bangladeshi, Whole genome sequencing, Single nucleotide variants, Gene Ontology, Genome mapping, NCBI SNP, NCBI Clinvar

## Abstract

**Objective:**

The major objective of the study was to sequence the whole genome of four Bangladeshi individuals and identify variants that are known to be associated with functional changes or disease states. We also carried out an ontology analysis to identify the functions and pathways most likely to be affected by these variants.

**Results:**

We identified around 900,000 common variants and close to 5 million unique ones in all four of the individuals. This included over 11,500 variants that caused nonsynonymous changes in proteins. Heart function associated pathways were heavily implicated by the ontology analysis; corroborating previous studies that claimed the Bangladeshi population as highly susceptible to heart disorders. Two variants were found that have been previously identified as pathogenic factors in familial hypercholesteremia and structural disorders of the heart. Other pathogenic variants we found were associated with pseudoxanthoma elasticum, cancer progression, polyagglutinable erythrocyte syndrome, preeclampsia, and others.

**Supplementary Information:**

The online version contains supplementary material available at 10.1186/s13104-021-05514-x.

## Introduction

The original Human Reference Genome had no representation from the subcontinent. Subsequently, the 1000 genome project [1, 2] added genome data from the region. Overall, the current database of human variants is still lacking in heavy representation from this region. The addition of more genomic and variation data from this region will improve our understanding of how different genetic markers and predispositions are distributed globally; especially since many countries across the world have a large Bangladeshi populations.

Here we undertake a pilot study using the whole genome sequences from four Bangladeshi individuals, labelled samples S1, S6, S19 and S21, to gain an understanding of the functionally relevant single nucleotide variations (SNVs) that can occur in this population. The primary goal of this study was to identify variants that can be associated with functional traits and disease states.

## Main text

### Materials and methods

#### Sequencing

This study was approved by the Ethical Committee under the National Institute of Cancer Research and Hospital, Mohakhali, Dhaka-1212, Bangladesh (Ref. No. NICRH/Ethics/2019/525, Date: 22.09.2019), consistent with the declaration of Helsinki-Ethical Principles, October 2008. DNA extraction and sequencing was carried out at Genome Research Laboratory, Bangladesh Council of Scientific and Industrial Research (BCSIR). The individuals chosen for this pilot study did not have any known underlying conditions or genetic disorders. A small aliquot (~ 5 ml) of blood sample was collected from each person and genomic DNA was extracted using Maxwell RSC whole blood DNA extraction kit (Promega) according to the manufacturer’s instructions. 300 ng gDNA from each of the four samples was used to prepare paired-end libraries with the Nextera™ DNA Flex Library Preparation kit with an average insert size of 600 bp. All manufacturer guidelines were followed (Illumina Inc., San Diego, CA). Sequencing was done using Novaseq 6000 sequencing platform.

#### Variant calling, annotation and analysis

Illumina Basespace Sequence hub, Dragon Germline 3.4.5 (DRAGEN Host Software Version 05.021.332.3.4.5 and Bio-IT Processor Version 0x04261818) was used for mapping the sequenced genomes with the human reference genome (GRCh38.p2) and the subsequent variant calling. The VCF files were annotated using Annovar [3]. These were subsequently compared with known human variation databases to identify the presence of functionally relevant mutations (for example pathogenic variants). Human variation datasets were obtained from the UCSC and NCBI repositories [4, 5]. Known variants were identified in our samples using bedtools [6] and manual filtering using R. Strand bias was accounted for using the Fisher Exact Test. This was carried out for each variant to determine if the concerned allele was supported by one strand more so than the other. Variants with scores of 0 or close to 0 were included in the subsequent analysis. Finally, we looked at the genomic locations of the exonic variants to list all the genes that contained these changes. The genes were then used to carry out an ontology search in order to identify the biological pathways and functions that are most likely to be effected as a result of these SNPs. This was done using DAVID, with all parameters set to default. The importance of these genes was also visualized using ReactomePA. This was done to highlight the major pathways associated with these genes. Briefly this gave us the pathways that are most likely to be impacted as a result of functional alterations in the genes that contained the aforementioned variants. Once more all parameters were kept at default.

### Results

The sequencing and mapping produced between 1.1 billion and 1.46 billion reads for each of the samples. Samples S1, S6 and S19 produced between 1.3 and 1.46 billion reads,; while for S21 the reads dropped down to around 1.1 billion. Under 25% of all reads were unmapped for all samples. The total number of reads aligned to the reference genome were 1387640908, 1498505945, 1387640908,1023927992 for samples S1, S6, S19 and S21, respectively. The coverage was 63 ×, 69 ×, 63 × and 47 × for S1, S6, S19, and S21 respectively (Additional file 1: Table S1).

All four samples contained between 5 million and 5.5 million variants. S1 gave 5,279,748 variants and after removing variants with QUAL < 20 the total number of variants came down to 5,000,704. Sample S6 had 5,345,421 variants initially and 5,064,885 after removing low quality calls. Sample S19 produced 5,269,076 variants with low quality calls and 4,970,655 calls without them. Lastly Sample S21 produced 5,260,335 calls with low quality variants and 4,966,352 variants after they had been filtered. Approximately 900,000 previously identified variants were found in each of the datasets.

Additional file 2: Figure S1 summarizes the variant calls. The QUAL scores were generally above the threshold of 20. The mapping qualities were mostly very high, with the highest peaks observed near 250. Read depth was concentrated mostly around 50 as an approximate median. The variant count per window summarizes the number of variants observed per each genomic interval of 1 kilobase pair (kbp) along the genome. The peak close to zero indicates most windows did not contain any variants (Additional file 2: Figure S1). This was visualized using the vcfR package on R [7].

The number of variants per chromosome correlated with chromosome size. Chromosome 1 had the most number of variants, averaging around 4.13 million for four samples, while Chromosome 22 had the least, averaging around 80,500. As for the base changes associated with the single nucleotide variants (SNVs), the four most common types of changes were to A to G, G to A, T to C, and C to T. Additional file 3: Figure S2 shows heatmaps of these four types of changes for each chromosome. The patterns are as expected with the larger chromosomes harboring more variants. The identical color band gradient of each chromosome for all four variant types indicates each chromosome contained near equal proportions of each type of base substitution.

The protein coding genes with the highest numbers of exonic variants were identical for all four samples. A total of 9702 genes harbored variants in all four samples. All individuals contained 25,000 exonic variants, of which 24,979 were common between all four (Additional file 4: supplementary files 1–4). The genes with the most variants were *MUC3A, MUC5AC, AHNAK2, MUC12, MUC4, FCGBP, MUC16, FLG, HLA A, HLA B* (Additional file 5: Tables S2A–D).

Among the exonic variants, 11,582 were nonsynonymous mutations, 12,296 were synonymous, 192 were nonframeshift insertions, 218 were nonframeshift deletions, 110 were stop-gain variants, 116 were frameshift deletions, 98 were frameshift insertions, 10 were stop-loss and 378 were unknown. Out of all the exonic variants, 9524 were homozygous variants in all four genomes.

**Fig. 1:**
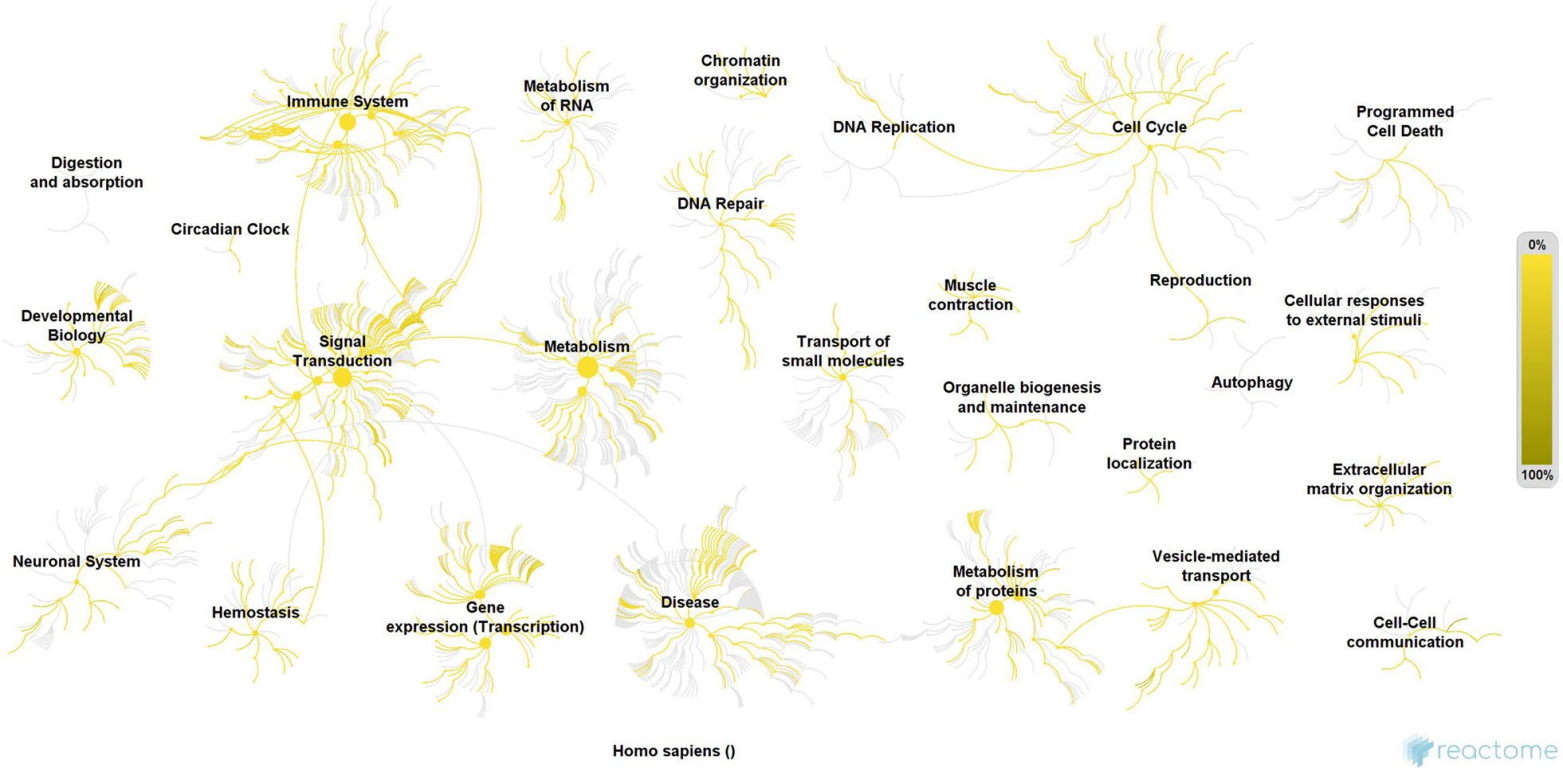
Visualization of metabolic pathways potentially affected by the highly variable genes in Bangladeshi individuals. Most notably, disease and immune system related pathways have been shaded in a darker yellow, indicating that a number of cascades and pathways whose dysregulation is connected to disease manifestations are likely to be impacted as a result of the genomic variants

Afterwards we investigated to the functional significance of these variants. The potential effects of these variants on different metabolic pathways was visualized using the Reactome online sever (Fig. 1). As it can be seen, the disease associated pathways are shaded in a darker yellow, suggesting that the genes containing the most variants are strongly connected to these pathways.

Afterwards we focused on the known clinical variants present in the genomes under investigation. A total of 3628 clinical variants were found in the Bangladeshi individuals. Seventeen of these were known pathogenic variants. 5 of these were associated with Pseudoxanthoma_elasticum. The rest of the implicated diseases were all represented by 1 variant each (Table 1).

**Table 1:** The diseases that are associated with the pathogenic variants found within our samples

Disease	Variants	Pathogenic
Pseudoxanthoma elasticum	5	5
Bardet-biedl syndrome 2/6, digenic	1	1
Cancer progression and tumor cell motility	1	1
Deafness, autosomal recessive 9	1	1
Diamond-Blackfan anemia_4	1	1
Encephalopathy,_progressive,_early-onset,_with brain edema_and/or_leukoencephalopathy	1	1
Familial hypercholesterolemia	1	1
Lynch syndrome	1	1
Polyagglutinable erythrocyte syndrome	1	1
Preeclampsia/eclampsia 4	1	1
Prekallikrein deficiency	1	1
Sandhoff disease,_infantile type	1	1
Serum amyloid a variant	1	1
Spastic paraplegia 75, autosomal recessive	1	1
Spongy degeneration of central nervous system	1	1
Structural heart defects and renal anomalies syndrome	1	1
Thyroxine-binding globulin, variant P	1	1

The NCBI Clinvar database was used to identify these previously known disease causing mutations. Two heart function associated disorders are implicated, which would seem to coincide with the relatively high incidence of heart diseases in Bangladesh. The most heavily implicated disease is Pseudonanthoma elasticum, for which the Bangladeshi individuals harbored 5 known pathogenic variants

**Fig. 2:**
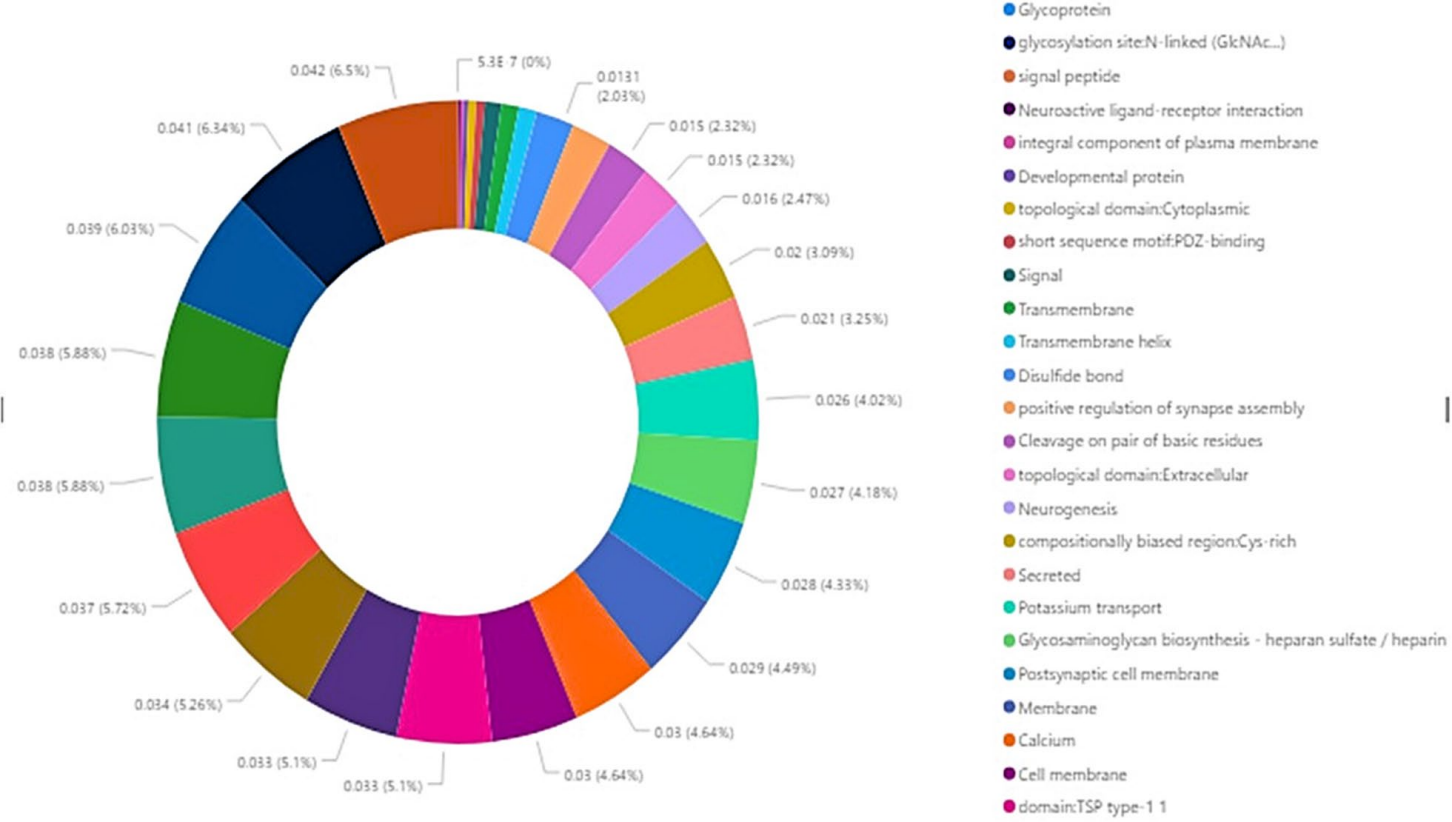
Functions most significantly affected by the genes which contained variants in the Bangladeshi individuals. Functions with Benjamini values more than the 0.05 threshold were omitted. The legend on the right only shows the 17 functions with the most significance (lowest scores)

In terms of functional importance, we found that a considerable number of our variants occurred in genes involved in cholesterol metabolism and cardiac functions, as per DAVID. Although adjusted p-values for significance were above 0.05 for all of these. However two of the aforementioned disease causing variants were directly associated with structural heart defects and hypercholesteremia respectively; suggesting the possibility that the variants occurring in heart function genes may be significant still. These two variants in question are the 11100236 G to A change in the LDLR gene in chromosome 19 and the 4 nucleotide deletion at position 56633141 of chromosome 14 in the TMEM260 gene. The functions that did have significant adjusted p-values or Benjamini scores of significance include glycosylation functions, signal peptidase functions, neuroactive ligand-receptor interactions, and glycoprotein associated functions; these were the five with the lowest Benjamini values (Additional file 5: Table S2A–D). Figure 2 displays this.

### Discussion

This study was the first preliminary step in a large scale population wide cataloguing of genomic features in the Bangladeshi population. Our primary focus was on the characterization of specific genetic signatures that impact various phenotypic features of Bangladeshi individuals. As part of this initial study involving four individuals, we identified a number of clinically relevant variants.

A number of genes containing significant numbers of variants in the Bangladeshi samples implicated various heart associated disorders when analyzed. The genes *CSMD1, EPHA3, and PTPRD* were all linked with heart or heart associated disorders by DAVID’s algorithm [8, 9]. Previous studies have often listed cardiovascular disease as one of the biggest causes of mortality in Bangladesh [10] and possible genetic links that may predispose the population to these conditions should be investigated further. A number of non-pathogenic clinically relevant variants were also identified in these genes; for example the chr6:121447564 G > A change which is classified as benign [11]. There were also a number of genes which only contained non-pathogenic variants in our samples but have been known to harbor pathogenic ones in other populations, such as *GABRD* and *SKI* (both known to contain pathogenic variants associated with heart diseases) [12, 13]. *SKI* contained two nonsynonymous substitutions in our samples.

The two heart disease associated pathogenic variants occurred in the low density lipoprotein receptor and the transmembrane protein 260. The former holds obvious importance with regards to cardiac health. In fact a number of mutations in this gene have been associated with familial hypercholesterolemia in other populations [14]. Furthermore, this protein is also believed to interact with *C. difficile* toxins and facilitate toxin entry into cells [15]. This is a pathogenic bacteria that causes infections of the gut and diarrhea. Such diseases have high incidence in Bangladesh, albeit caused by other agents such as *Vibrio cholerae*. It is interesting to speculate whether the presence of these variants can also make individuals more vulnerable to other gut infection causing microbes such as Vibrio cholerae. The variants associated with Pseudoxanthoma_elasticum also hold potential significance in this regard. One of the major clinical manifestations of this genetic disorder is atherosclerosis; providing yet more evidence of genetic predisposition to heart conditions [16].

Finally, we identified 28 genes with previously unidentified unique mutations. Most of these genes were associated with alternative splicing and other functions connected with polymorphisms in the DNA/RNA (Additional file 6: Table S3A, and 3B).

Although we have used four individuals in this study, the genome analysis of these individuals have provided many known and unknown variants which may have an impact on the health of these individuals as well as the broader Bangladeshi population. Additionally, as we add more subjects to this initiative, we will then be able identify and validate variants that are unique to the Bangladeshi population, as well the resultant genetic predispositions.

## Limitations

The major limitation of this study was the small sample size and the consequent limited analysis, especially in finding the disease-variant correlation. While we are confident in the fact that our major findings are the clinical variants that have been known to cause diseases in other populations, thus rendering the possibility of these variants arising as a result of sequencing error very small, a larger sample size would nonetheless add more validation. The findings listed and discussed here, along with the data available in the additional files can be used to focus genome research endeavors in the future.

## Supplementary Information

**Additional file 1: Table S1.** Sequencing related statistics for all four samples; including number of reads, number of mapped reads, number of unmapped reads, etc.

**Additional file 2: Figure S1**. Variant Summary and Statistics.jpg: Summary of variant calls for all four samples. Samples 1, 19 and 21 show almost identical trends. Whereas sample 6 appears to differ with regards to the read depth, showing only two peaks around the 50 mark, compared to the other three which had three or four. QUAL scores were mostly concentrated in the 20–100 window, though most variants passed the quality threshold of 20. Mapping quality was generally high for most of our calls in all four samples, while the variant count per window shows that most 1 kbp windows did not contain any variants, indicating variants were concentrated in particular regions of the genome, as is expected for most genomes

**Additional file 3: Figure S2**: Heatmap of Base Substitutions.jpg: Heatmap showing the distribution of the four most common single nucleotide changes for all four samples. Chromosomes 1 and 2 were white, indicating the highest numbers of variants occur in these two chromosomes. Red indicates the lowest numbers of variants, as can be seen with chromosomes 21, 22 and Y. All chromosomes show a uniform colour band which also suggests that each of the four variant types occur in equal numbers for all chromosomes. This would support the general logic dictating the distribution of SNPs in that they are random and correlate with the size of the genomic region in question.

**Additional file 4.** Supplementary file 1: 1.exonic_variant_function, Supplementary File 2: 6.exonic_variant_function, Supplementary File 3: 19.exonic_variant_function, Supplementary File 4: 21.exonic_variant_function. Full list of exonic variants for each sample, along with associated statistics for each variant (1-Sample 1, 2-Sample 6, 3-Sample 19, 4-Sample 21).

**Additional file 5: Table S2A-2D**: Functions most likely to be effected by the genes which contained variants (2A-Sample 1, 2B-Sample 6, 2C-Sample 19, 2D-Sample 21).

**Additional file 6: Table S3 A:** List of genes which contained exonic variants for all our samples. **Table S3 B**. Functions Effected by Unique Variants.csv: Functions effected by genes which contained unique/previously undiscovered exonic variants in our samples.

## Data Availability

The data supporting the conclusions of this article are included within the article. Raw Sequence data for four samples are available under the SRA accession number: SAMN14089716, SAMN14089717, SAMN14089718, and SAMN140897197 (https://www.ncbi.nlm.nih.gov/biosample?LinkName=bioproject_biosample_all&from_uid=606337).

## Abbreviations

SNP: Single nucleotide polymorphism
SNV: Single nucleotide variant
S1: Sample/Individual 1
S6: Sample/Individual 6
S19: Sample/Individual 19
S21: Sample/Individual 21
BCSIR: Bangladesh Council of Scientific and Industrial Research
VCF: Variant Call Format
NCBI: National Center for Biotechnology Information
Kbp: Kilobase pair

## References

[1] The 1000 Genomes Project Consortium (2012). An integrated map of genetic variation from 1092 human genomes. Nature.

[2] The 1000 Genomes Project Consortium (2010). A map of human genome variation from population-scale sequencing. Nature.

[3] Wang K, Li M, Hakonarson H (2010). ANNOVAR: functional annotation of genetic variants from high-throughput sequencing data. Nucleic Acids Res.

[4] Karolchik D (2004). The UCSC Table Browser data retrieval tool. Nucleic Acids Res.

[5] Sherry S (2001). dbSNP: the NCBI database of genetic variation. Nucleic Acids Res.

[6] Quinlan A (2014). BEDTools: The Swiss-Army tool for genome feature analysis. Curr Protoc Bioinform..

[7] Knaus BJ, Grünwald NJ. 2016 VcfR: a package to manipulate and visualize VCF format data in R. 10.1111/1755-0998.12549 27401132

[8] Cunningham F, Achuthan P, Akanni W, Allen J, Amode M, Armean I (2018). Ensembl 2019. Nucleic Acids Res.

[9] Huang D, Sherman B, Lempicki R (2008). Systematic and integrative analysis of large gene lists using DAVID bioinformatics resources. Nat Protoc.

[10] Chowdhury R, Alam D, Fakir I, Adnan S, Naheed A, Tasmin I (2015). The Bangladesh risk of acute vascular events (BRAVE) study: objectives and design. Eur J Epidemiol.

[11] Landrum M, Lee J, Benson M, Brown G, Chao C, Chitipiralla S (2017). ClinVar: improving access to variant interpretations and supporting evidence. Nucleic Acids Res.

[12] Doyle AJ, Doyle JJ, Bessling SL, Maragh S, Lindsay ME, Schepers D (2012). Mutations in the TGF-β repressor SKI cause Shprintzen-Goldberg syndrome with aortic aneurysm. Nat Genet.

[13] Miller DT, Adam MP, Aradhya S, Biesecker LG, Brothman AR, Carter NP (2010). Consensus statement: chromosomal microarray is a first-tier clinical diagnostic test for individuals with developmental disabilities or congenital anomalies. Am J Hum Genet.

[14] Jensen H, Jensen L, Meinertz H, Hansen P, Gregersen N, and Færgeman O. Spectrum of LDL receptor gene mutations in Denmark: implications for molecular diagnostic strategy in heterozygous familial hypercholesterolemia. Atherosclerosis, 1999;146(2), pp.337-344. https://www.atherosclerosis-journal.com/article/S0021-9150(99)00158-6/abstract. 10.1016/s0021-9150(99)00158-6 10532689

[15] Tao L, Tian S, Zhang J, Liu Z, Robinson-McCarthy L., Miyashita S, et al. 2019. Sulfated glycosaminoglycans and low-density lipoprotein receptor contribute to Clostridium difficile toxin A entry into cells. Nature Microbiology, 4(10), p. 1760-1769. https://www.nature.com/articles/s41564-019-0464-z. 10.1038/s41564-019-0464-z PMC6754795 31160825

[16] Finger R, Issa P, Ladewig M, Götting C, Szliska C, Scholl H (2009). Pseudoxanthoma elasticum: genetics, clinical manifestations and therapeutic approaches. Surv Ophthalmol.

