# Supplementary figures and images for: Whole genome mapping and identification of single nucleotide polymorphisms of four Bangladeshi individuals and their functional significance
Source: BMC Res Notes. 2021 Mar 20;14:105. doi: 10.1186/s13104-021-05514-x (PMC7981821; doi:10.1186/s13104-021-05514-x)

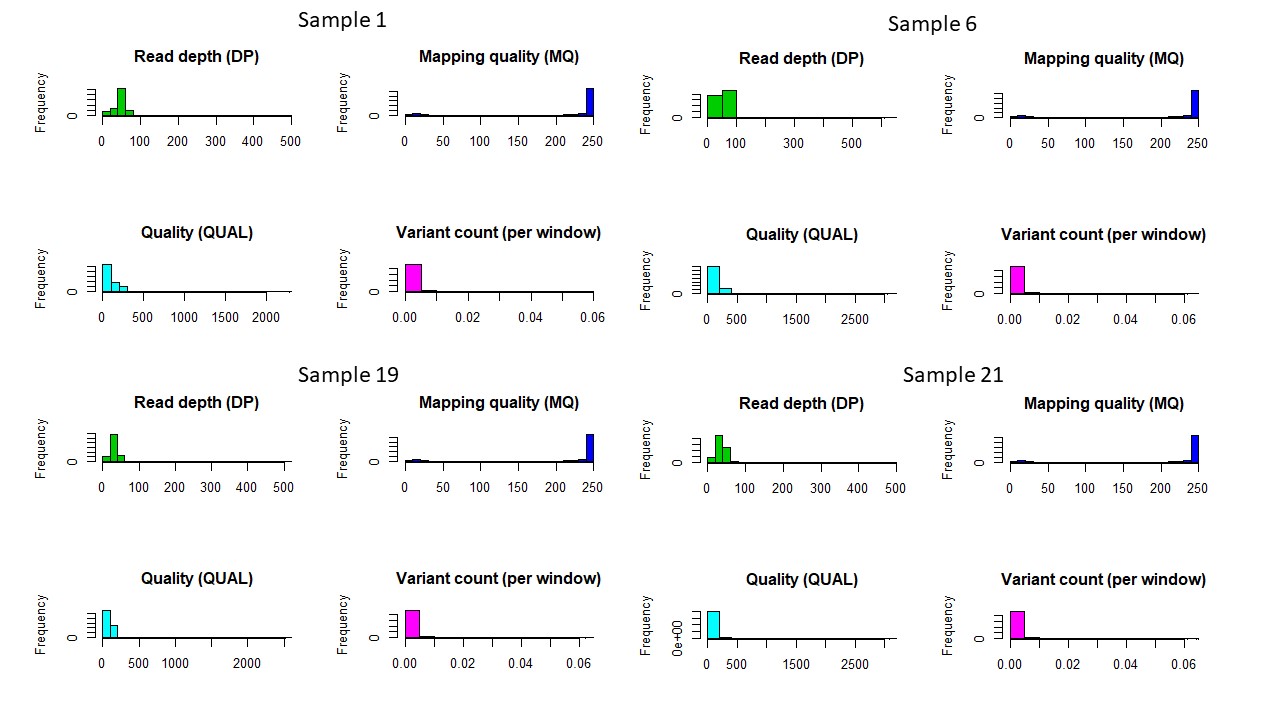

Supplement: Supplementary file 2 — Additional file 2: Figure S1. Variant Summary and Statistics.jpg: Summary of variant calls for all four samples. Samples 1, 19 and 21 show almost identical trends. Whereas sample 6 appears to differ with regards to the read depth, showing only two peaks around the 50 mark, compared to the other three which had three or four. QUAL scores were mostly concentrated in the 20–100 window, though most variants passed the quality threshold of 20. Mapping quality was generally high for most of our calls in all four samples, while the variant count per window shows that most 1 kbp windows did not contain any variants, indicating variants were concentrated in particular regions of the genome, as is expected for most genomes [file 13104_2021_5514_MOESM2_ESM.jpg]

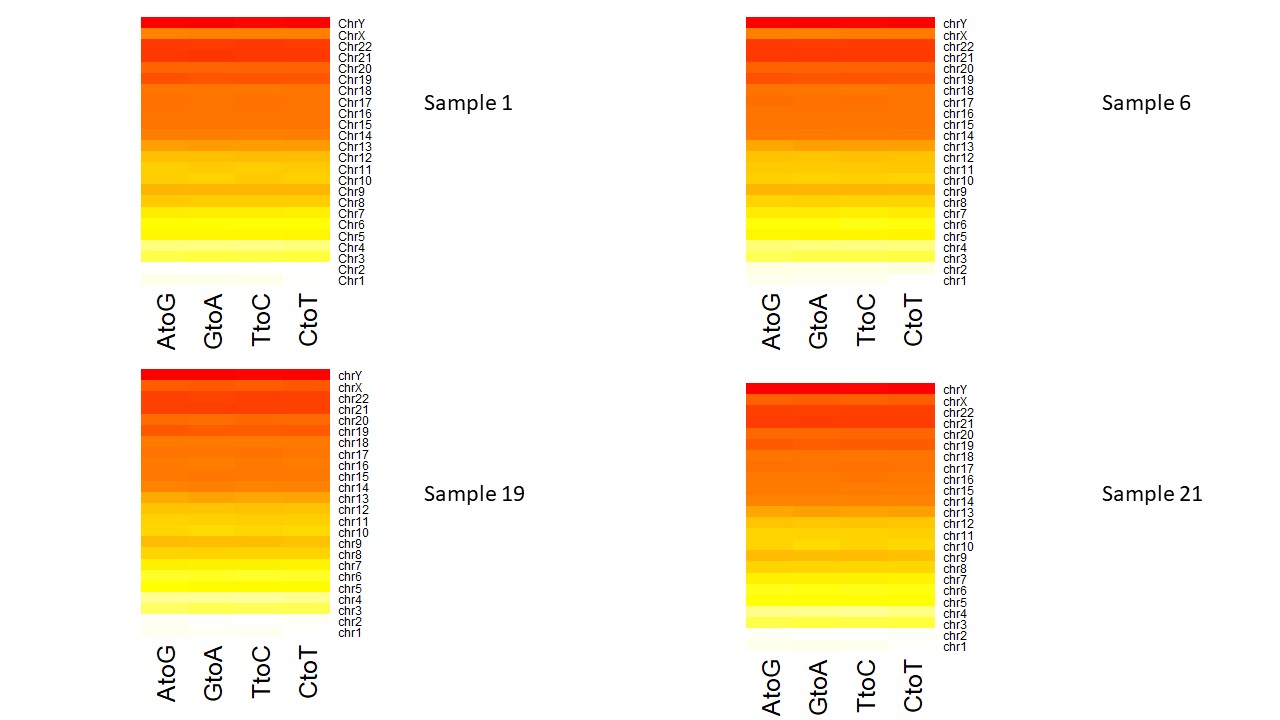

Supplement: Supplementary file 3 — Additional file 3: Figure S2: Heatmap of Base Substitutions.jpg: Heatmap showing the distribution of the four most common single nucleotide changes for all four samples. Chromosomes 1 and 2 were white, indicating the highest numbers of variants occur in these two chromosomes. Red indicates the lowest numbers of variants, as can be seen with chromosomes 21, 22 and Y. All chromosomes show a uniform colour band which also suggests that each of the four variant types occur in equal numbers for all chromosomes. This would support the general logic dictating the distribution of SNPs in that they are random and correlate with the size of the genomic region in question. [file 13104_2021_5514_MOESM3_ESM.jpg]
